# Cardiac Regeneration using Growth Factors: Advances and Challenges

**DOI:** 10.5935/abc.20160097

**Published:** 2016-09

**Authors:** Juliana de Souza Rebouças, Nereide Stela Santos-Magalhães, Fabio Rocha Formiga

**Affiliations:** 1 Laboratório de Imunopatologia Keizo-Asami - Universidade Federal de Pernambuco (UFPE), Recife, PE - Brazil; 2 Programa de Pós-Graduação em Biologia Celular e Molecular Aplicada - Universidade de Pernambuco (UPE), Recife, PE - Brazil; 3 Curso de Pós-Graduação em Patologia (UFBA/FIOCRUZ) - Centro de Pesquisas Gonçalo Moniz, Fundação Oswaldo Cruz (FIOCRUZ), Salvador, BA - Brazil

**Keywords:** Myocardial Infarction, Myocardial Ischemia, Vascular Remodeling, Intercellular Signaling Peptides and Proteins, Cell-and Tissue Based Therapy

## Abstract

Myocardial infarction is the most significant manifestation of ischemic heart disease and is associated with high morbidity and mortality. Novel strategies targeting at regenerating the injured myocardium have been investigated, including gene therapy, cell therapy, and the use of growth factors. Growth factor therapy has aroused interest in cardiovascular medicine because of the regeneration mechanisms induced by these biomolecules, including angiogenesis, extracellular matrix remodeling, cardiomyocyte proliferation, stem-cell recruitment, and others. Together, these mechanisms promote myocardial repair and improvement of the cardiac function. This review aims to address the strategic role of growth factor therapy in cardiac regeneration, considering its innovative and multifactorial character in myocardial repair after ischemic injury. Different issues will be discussed, with emphasis on the regeneration mechanisms as a potential therapeutic resource mediated by growth factors, and the challenges to make these proteins therapeutically viable in the field of cardiology and regenerative medicine.

## Introduction

Cardiovascular diseases (CVD) are the leading cause of death among men and women worldwide, in all racial and ethnic groups.^1^ In the United States, these diseases account for approximately 57% of all deaths in the country.^2^ In Europe, CVD cause 4.3 million deaths every year, which represents almost half (48%) of all deaths in that continent.^3^ CVD are also the major death cause in Brazil, with a specific mortality rate for ischemic heart diseases of 53.8 deaths for every 100,000 inhabitants.^4^

In the CVD group, coronary artery disease (CAD) and peripheral artery disease (PAD) are significant causes of morbidity and mortality, requiring surgical bypass procedure or angioplasty for thousands of patients. On the other hand, myocardial infarction (MI) is the most important manifestation of ischemic heart disease and is also associated with high morbidity and mortality. Ischemia is responsible for cardiac muscle damage, including the loss of cardiomyocytes. This process leads to a negative cardiac remodeling causing the cardiac tissue with a normal contractile function to be replaced by a non-functional scar tissue. The myocardium then produces a compensatory hypertrophic mechanism against ischemia-induced wound healing. However, the hypertrophy may make the heart susceptible to the onset of arrhythmias, ventricular fibrillation and massive heart attack.^5,6^ Although advanced revascularization procedures (angioplasty, catheterization, bypass) have contributed to a marked reduction in mortality for CVD, a significant number of patients are not eligible to these procedures or achieve incomplete revascularization with these interventions. Consequently, many of these patients show persistent symptoms of cardiac ischemia despite intensive medical care. They probably suffer from severe diffuse atherosclerotic disease, which cannot be treated by surgery or angioplasty. Symptomatic obstructive vascular disease leads to claudication, peripheral ischemia, angina and congestive heart failure, significantly limiting the quality of life of these patients.

Treatment of MI includes the use of drugs (antiplatelet agents, oral anticoagulants, nitrates, β-adrenergic blockers, ACE inhibitors, and others), surgical reperfusion and revascularization procedures, and, in more complex cases, heart transplantation. In the past decade, there was growing investigation on new strategies for regeneration of the injured myocardium, including gene therapy,^7,8^ cell therapy,^9,10^ and the use of growth factors.^11^ The later has also been investigated for the induction of therapeutic angiogenesis for peripheral arterial disease.^12^

The use of growth factors has aroused interest in cardiovascular medicine because of the direct action of these factors on several cell functions such as adhesion, proliferation, migration, and others. When obstruction of the coronary artery flow occurs, induction of angiogenesis by growth factors represents an important mechanism of myocardial repair and protection under hypoxic conditions, resulting in the formation of new vessels.^13^ Consequently, tissue perfusion increases, ultimately leading to a better cardiac function.

On the other hand, the regenerative potential of growth factors has gained great importance in the context of cell therapy. Studies have demonstrated that the benefits derived from the administration of stem cells in the infarct area result, to a greater extent, from the paracrine effect of the growth factors secreted by the cells implanted than from the direct action of the cells in the infarct tissue.^9,14-16^ These factors show the potential of inducing different regeneration mechanisms: positive remodeling of the extracellular matrix, proliferation of adult cardiomyocytes, recruiting/homing of cardiac stem cells, antiapoptotic and/or angiogenic effect.^11,17^ Together, these mechanisms may reduce inflammation, fibrosis and inadequate perfusion of the ischemic myocardium, promoting tissue repair and improvement of the cardiac function.^9^

Despite the mechanisms of growth-factor-induced tissue regeneration, the therapeutic potential of these proteins is limited by their short biological half-life, low plasma stability and low specificity to target organs. In fact, Hwang and Kloner administered a cocktail of growth factors in rats intraperitoneally and did not observe benefits in the cardiac function, reduction of the infarct size or increase in vascularization.^18^

Thus, the clinical use of growth factors depends on new formulation technologies able to increase their half-lives, keep their bioactivity, and control their local delivery in target tissues. In this context, micro- and nanostructured systems have been used as delivery platforms,^19,20^ and are a promising formulation strategy for the therapeutic use of growth factors for cardiac regeneration.^11^

The objective of this review is to address the strategic role of growth factor therapy for cardiac regeneration, considering its innovative and multifactorial character on cardiac repair after an ischemic injury.

### Mechanisms of cardiac regeneration

The innate capacity of the human heart for self-regeneration is not enough to compensate the loss of cardiac muscle after an ischemic injury.^9^ Unlike what is observed with skeletal muscles, in which satellite cells and myoblasts form new myocytes a few days after an injury, cardiomyocytes from the border zone of the infarct rarely divide after an ischemic event.^21^ In a lesion induced by infarct, the heart loses approximately 50 g of muscle, and this can result in the death of 2 billion cardiomyocytes.^22,23^ This myocardial aggression triggers and modulates tissue reparative changes, including dilatation, hypertrophy, and formation of a collagen scar.^24^ In relation to cell renewal, the mechanisms of endogenous repair are not enough to induce significant renewal of the muscle mass lost after the ischemic injury.

Cardiomyocyte proliferation plays a key role in cardiac regeneration in some vertebrates, but the proliferative capacity of these cells is limited in the adult hearts of mammals.^21^ Another potential cell renewal mechanism is the mobilization of progenitor cells from the bone marrow to the ischemic area and their differentiation into functional cardiomyocytes.^9^ However, mobilization and homing of these progenitors are also not enough to induce significant regeneration. The myocardium also shelters a population of resident cardiac stem cells (CSC) with potential to differentiate into cardiomyocytes.^25,26^ The CSC seem to account for the baseline turnover of cardiomyocytes. However, this renewal probably occurs at very low rates in the absence of lesion.^27^

The efficacy of these endogenous mechanisms of tissue repair is limited by the hostile microenvironment of the infarcted myocardium, which is characterized by ischemia, inflammation, fibrosis and inadequate angiogenesis. This microenvironment probably prevents, the CSC activation. On the other hand, excessive inflammation also prevents progenitors mobilization and homing. The formation of fibrotic tissue is necessary to prevent muscle rupture after infarction, but the high level of fibrosis represents an important physical barrier to myocardial cell regeneration.^9^ Therefore, mitigation of this hostile environment should contribute to cardiac repair, especially the reduction of local inflammation, apoptosis and fibrosis, as well as the increase in vascularization in the infarct and peri-infarct areas.

### Growth factors inducing regenerative mechanisms

Angiogenesis refers to the development of blood vessels from a pre-existing vascular bed. From the medical point of view, the objective is to stimulate vessel growth in patients with conditions characterized by insufficient blood flow, such as ischemic heart diseases and peripheral vascular diseases.^28^

As regards the latter aspect, the identification of growth factors that induce the angiogenic process stimulated the interest in the use of these proteins for the induction of therapeutic angiogenesis.^11^ In the case of myocardial infarction, angiogenic therapy with growth factors may salvage the ischemic tissue at early stages of infarction, by supplying the tissue with new vessels. This process is essential to prevent heart failure through the control of cardiomyocyte hypertrophy and contractility.^29^ In fact, angiogenesis is the main growth factor-induced reparative mechanism and has been the mechanism most often investigated in experimental studies and clinical trials on injured myocardium repair. Most of these studies have dedicated their efforts toward the angiogenic and regenerative potential of vascular endothelial growth factor (VEGF)^30-33^ and fibroblast growth factor (FGF).^31,34-36^

Mitigation of the ischemic injury in the cardiac tissue may be induced by antiapoptotic factors, which exert potentially cardioprotective effects. Hepatocyte growth factor (HGF) was first identified as a hepatocyte mitogen, with chemotactic and antiapoptotic actions in different cell types.^37^ In rats undergoing ischemia and reperfusion, intravenous administration of HGF reduced apoptosis in cardiomyocytes and the infarct size.^38^ Other antiapoptotic factors with therapeutic potential in cardiac regeneration include platelet-derived growth factor (PDGF-BB)^39^ and protein thymosin β4^40^, IL-11^41^, IL-33^42^, and others.

Endogenous mechanisms mediated by progenitors and stem cells include mobilization and homing of bone marrow progenitors as well as CSC activation. These cells may differentiate into new cardiomyocytes after the ischemic injury, but their number is reduced or they are insufficiently activated to produce significant muscular regeneration. Some proteins show the potential to mobilize bone marrow progenitors to the cardiac lesion area or activate CSC. These properties may be therapeutically explored as regenerative mechanisms activated by growth factors or recombinant proteins, such as the granulocyte colony stimulating factor (G-CSF),^43^ HGF,^44^ stromal cell-derived factor (SDF-1),^45^ and others.

The paradigm of the heart as a completely differentiated organ was contested based on the identification of mitogens able to induce adult cardiomyocytes to enter into the cell cycle.^46,47^ This process opens the possibility to stimulate a new regeneration mechanism in the infarcted heart, leading to the formation of a population of new cardiomyocytes capable of replacing the cell mass lost due to the ischemic injury. Three extracellular factors have been identified for their ability to activate receptors involved in cardiomyocyte proliferation: acidic fibroblast growth factor (FGF-1),^48^ neuregulin (NRG-1),^47^ and periostin.^49^ Treatment of infarcted rats with FGF-1 in combination with a mitogen-activating protein kinase (MAPK) p38 resulted in increased cardiomyocyte mitosis and improved cardiac function.^50^ Studies have demonstrated improved cardiac function in infarcted mice treated with daily injections of NRG-1.^47,51^ A summary of growth factor-induced cardiac regeneration mechanisms is shown in Table 1.

**Table 1:** Main growth factors inducing the mechanisms of cardiac regeneration

Factor	Mechanisms	Reference
VEGF	Angiogenesis	30-33
FGF	Angiogenesis	31,34-36
HGF	Antiapoptosis	37,38, 44
	CSCs chemotaxis	
SDF-1	Hematopoietic stem cells mobilization and homing	45
IGF-1	Stem cells and progenitor cells viability and differentiation	52
PDGF	Antiapoptosis	39
G-CSF	Antiapoptosis	43
	Hematopoietic stem cells mobilization and homing	
Intermedin	Angiogenesis	53
Angiopoietin	Angiogenesis, remodeling and vascular stabilization	54
Periostin	Cardiomyocyte proliferation	49
Neuregulin-1	Cardiomyocyte proliferation	47
Erythropoietin	Antiapoptosis	55

VEGF: vascular endothelium growth factor isoforms; FGF: fibroblast growth factor; HGF: Hepatocyte growth factor; SDF-1: stromal cell-derived factor; IGF‑1: Insulin‑like growth factor 1; PDGF: platelet-derived growth factor; G-CSF: granulocyte colony stimulating factor.

### Challenges in growth factor formulation

In the past two decades, intensive research on the mechanisms of cardiac regeneration has resulted in considerable advances in the discovery of therapeutic targets related to several growth factors. These proteins have been evaluated in experimental studies and clinical trials, which have demonstrated the safety and potential efficacy of these factors in the treatment of ischemic heart diseases, particularly myocardial infarction.^11,56^ However, an important challenge for establishing protein therapy for these diseases is the development of formulation technologies capable of ensuring the reparative mechanisms of these biomolecules and making them clinically viable.

Aspects related to dosage, route of administration, protein stability and biocompatibility should be considered. The ability of these formulations to incorporate multiple factors also represents a critical issue, considering the multifactorial character of the mechanisms involved in myocardial repair following ischemia. Together, these aspects have been previously reviewed and should guide the rational development of growth factor formulations for protein and/or cell therapy focusing on cardiac generation.^11^

Micro- and nanostructured controlled delivery systems show several advantages over conventional formulations that deliver biopharmaceuticals in their free form, usually in an aqueous vehicle for intravenous administration. By permitting a more adequate pharmacokinetic profile to the effects of the active compound, micro- and nanoformulations facilitate patient's adherence to treatment; provide protection to the active ingredient against enzymatic degradation; permit specific targeting to an organ or target-structure; local and controlled delivery of the molecule of interest. Polymeric systems (hydrogels, scaffolds, micro- and nanoparticles)^11,57,58^ and lipid systems (liposomes, solid lipid nanoparticles)^59,60^ have been used as cardiac delivery platforms of growth factors, which can be obtained from natural biomaterials (collagen/gelatin, fibrin, hyaluronic acid, alginate, chitosan, etc.) and synthetic materials (polyesters, amino acid polymers, polyacrylamide derivatives, and others).^11^

Polyesters such as poly (lactic acid-co-glycolic acid, PLGA) and polycaprolactone (PCL) are polymers approved for the use in drug delivery systems because of their low immunogenic potential and adequate biodegradation profile. Previous studies have demonstrated the biocompatibility of PLGA microparticles with the cardiac tissue and the efficacy of these particles as delivery systems of VEGF in the experimental treatment of myocardial infarction.^58,61^ Recently, Formiga and colleagues have demonstrated the efficacy of these microparticles as cardiac delivery systems of FGF-1 and NRG-1, ensuring the regenerative effects of these factors in an rat myocardial infarction model.^62^

### Perspectives

Future perspectives for the use of cardioregenerative factors are related to the development of new formulation technologies combined with smart, biocompatible, non-invasive materials. These advances should work as multifunctional structures that combine therapeutic and diagnostic functions in a single micro- or nanostructurated. Additionally, they will allow specific ligand-guided targeting on the material surface. The translational potential of these technologies is predictable, considering the diversity of growth factor-induced regeneration mechanisms. These processes should be explored with more clinical interest both as protein therapy and as adjuvant in stem-cell therapy for cardiac regeneration.
